# Neural Mechanisms of Attentional Switching Between Pain and a Visual Illusion Task: A Laser Evoked Potential Study

**DOI:** 10.1007/s10548-017-0613-8

**Published:** 2017-12-19

**Authors:** Andrej Stancak, Nicholas Fallon, Alessandra Fenu, Katerina Kokmotou, Vicente Soto, Stephanie Cook

**Affiliations:** 1 Department of Psychological Sciences, University of Liverpool, Liverpool, L69 7ZA UK; 2 Institute for Risk and Uncertainty, University of Liverpool, Liverpool, UK

**Keywords:** EEG, P2, Distraction analgesia, Source dipole model, Single-trial analysis

## Abstract

Previous studies demonstrated that pain induced by a noxious stimulus during a distraction task is affected by both stimulus-driven and goal-directed processes which interact and change over time. The purpose of this exploratory study was to analyse associations of aspects of subjective pain experience and engagement with the distracting task with attention-sensitive components of noxious laser-evoked potentials (LEPs) on a single-trial basis. A laser heat stimulus was applied to the dorsum of the left hand while subjects either viewed the Rubin vase-face illusion (RVI), or focused on their pain and associated somatosensory sensations occurring on their stimulated hand. Pain-related sensations occurring with every laser stimulus were evaluated using a set of visual analogue scales. Factor analysis was used to identify the principal dimensions of pain experience. LEPs were correlated with subjective aspects of pain experience on a single-trial basis using a multiple linear regression model. A positive LEP component at the vertex electrodes in the interval 294–351 ms (P2) was smaller during focusing on RVI than during focusing on the stimulated hand. Single-trial amplitude variations of the P2 component correlated with changes in Factor 1, representing essential aspects of pain, and inversely with both Factor 2, accounting for anticipated pain, and the number of RVI figure reversals. A source dipole located in the posterior region of the cingulate cortex was the strongest contributor to the attention-related single-trial variations of the P2 component. Instantaneous amplitude variations of the P2 LEP component during switching attention towards pain in the presence of a distracting task are related to the strength of pain experience, engagement with the task, and the level of anticipated pain. Results provide neurophysiological underpinning for the use of distraction analgesia acute pain relief.

## Introduction

Pain has been shown to be reduced while attention is directed to a stimulus occurring in a different sensory modality or consumed in an engaging cognitive task (Miron et al. 1989). Although earlier studies pointed to certain limitations in effects of distraction on pain intensity (Leventhal 1992; McCaul et al. 1992), the phenomenon of distraction-induced analgesia proved to be robust enough to alleviate acute procedural pain using video games (Seyrek et al. 1984), immersive virtual reality (Hoffman et al. 2011, 2004b), or watching TV (Bellieni et al. 2006).

Since pain signals potential or actual tissue damage, it easily captures attention and therefore disrupts ongoing cognitive or sensory processing (Eccleston and Crombez 1999). Balancing pain experience with concurrent cognitive or sensory activities requires a switch mechanism which operates automatically on a scale of hundreds of milliseconds and also takes into account instantaneous demands and motivational values of parallel tasks. The time interval following switching attention towards pain has been suggested to allow the background pain to invade the conscious mind and disrupt the cognitive performance further in chronic pain patients (Attridge et al. 2016; Vlaeyen et al. 2016). Pain intensity and performance in a distracting task have been shown to interact in a dose-dependent manner with the largest pain reduction and the largest disruption of performance occurring at the highest levels of both (Romero et al. 2013).

Previous fMRI studies, reviewed recently in Torta et al. (2017), pointed to a network of regions associated with pain reduction during attentional distraction, such as anterior cingulate cortex (Bantick et al. 2002; Buffington et al. 2005), anterior insula (Peyron et al. 1999), and thalamus and somatosensory cortex (Hoffman et al. 2004a). Focusing attention to the location of a noxious stimulus or pain unpleasantness has been shown to activate different brain networks known as medial and lateral pain system (Kulkarni et al. 2005). More recently, Kucyi et al. (2013) demonstrated that a salience network featured by anterior insula, dorsolateral prefrontal cortex and temporal-parietal junction were active when subjects spontaneously allocated larger attention to pain than to unrelated thoughts. However, BOLD-fMRI recordings cannot resolve brain activation patterns on a scale of hundreds of milliseconds which is the time scale at which instantaneous switching between pain and a parallel cognitive process would occur. Electroencephalographic LEPs, offering a temporal resolution on a scale of milliseconds, have been employed to analyse the cortical spatio-temporal patterns associated with attentional modulation of pain. Distraction of attention compared to focusing on pain has been shown to decrease the LEP components at centro-parietal midline electrodes in the latency interval of N2 and more often of the P2 component (Beydoun et al. 1993; Boyle et al. 2008; Friederich et al. 2001; García-Larrea et al. 1997; Kanda et al. 1996; Ohara et al. 2004; Schlereth et al. 2003; Siedenberg and Treede 1996). Positive centro-parietal components at latencies longer than 300 ms also encoded novelty and saliency effects in attentional oddball experiments (Legrain et al. 2003, 2002, 2009a; Siedenberg and Treede 1996; Zaslansky et al. 1996). The positive P2 LEP component reflects salience and novelty of noxious stimuli, and is also affected by the amount of cognitive load associated with a distracter (Legrain et al. 2012).

Allocation of attentional resources to pain in the presence of a goal-directed activity can be viewed as a dynamic interplay of the automatic, stimulus-driven, bottom up processes and goal-directed, intentional, top-down processes (Legrain et al. 2012, 2009b; Torta et al. 2017). Formation of pain experience reflects perceptual decision making in which prior information, such as anticipated pain intensity, plays a role (Wiech et al. 2014). To understand the rules and neural mechanisms which determine how pain experience changes during attentional distraction, in which both the pain experience and the engagement with the cognitive task vary over time, a single-trial analysis of subjective responses, task performance data, and cortical response is required.

Perceptual and affective outcomes of noxious stimuli can be viewed as functions of anticipated and perceived pain intensity. Aversive prediction error has been shown to affect fMRI responses to noxious stimuli in a learning paradigm (Roy et al. 2014). One of the electrophysiological manifestations of perceptual decision making is the feedback-related negativity (Gehring and Willoughby 2002), a negative subtraction potential occurring about 250–350 ms after the presentation of an outcome. While feedback-related negativity has been mostly linked with the reward prediction error in monetary tasks, stimuli signalling pain omission also produce feedback-related negativity similar to that occurring during monetary losses (Talmi et al. 2013). Electrophysiological studies involving prediction coding of aversive stimuli suggested that the salience aspect of the sensory stimulus associated with unexpected omission of a stimulus contributed to the feedback-related negativity potential independently of its hedonic value (Garofalo et al. 2014; Talmi et al. 2013). Notably, feedback-related negativity is a subtraction potential receiving its negative sign by subtracting a large positive potential over central-parietal midline region of the scalp in gains from that in losses. As the P2 component of LEPs reflects the salience aspect of a noxious stimulus (Legrain et al. 2010, 2012), it is possible that the salience of said stimulus results from a comparison of anticipated and perceived pain intensity. Therefore, we decided to also analyse whether trial-by-trial variations in the attention-sensitive LEP component would be related to the intensity prediction error which was evaluated on a single-trial basis as the difference between anticipated and perceived pain intensity.

Pain is a multifaceted, multidimensional experience believed to involve a sensory-discriminative, motivational-affective, and cognitive-evaluative dimension (Melzack and Casey 1968). We have recently analysed the dimensionality of subjective pain experience associated with a brief noxious laser stimulus and the spatio-temporal LEP patterns representing dimensions of the pain experience (Stancak et al. 2015). Five factors of subjective pain experience were extracted (factors representing essential aspects of pain, warming and after-sensations, temporal aspects of stimulus occurrence, body sensations, and anticipated pain). Four of these factors correlated with specific LEP components. While the decrease of pain intensity and unpleasantness is a well-established outcome in distraction analgesia, little is known about whether other aspects of pain experience besides pain intensity change during attentional distraction. Miron et al. (1989) found a decreased discrimination between noxious thermal stimuli during attentional distraction suggesting that diverting attention away from pain reduces the capacity to capture subtle aspects of pain. In contrast, the accuracy of spatial localisation of laser stimuli has been shown to be intact in the presence of distracting acoustic noise (Boyle et al. 2008).

To shed light on effects of attentional distraction in different aspects of pain experience, and to investigate whether attention-related changes in subjective pain experience would be manifested in trial-by-trial variations of the P2 LEP component, we decided to correlate the amplitudes of attention-sensitive LEP components with individual dimensions of pain experience on a single-trial basis. We employed a multiple linear regression analysis (Ratcliff et al. 2009; Rousselet et al. 2011) in which factors representing individual dimensions of pain experience and a measure of task engagement were used as predictors, and single-trial amplitude variations of the attention-sensitive LEP component (P2 potential) as the dependent measure. In this exploratory study, we predicted that the amplitude of the P2 component would be positively correlated with essential aspects of pain experience, featured by pain intensity, and negatively with the level of engagement with the distracting task. It was also hypothesised that trial-by-trial variations in amplitude of the P2 component would be correlated with the intensity prediction error in such a way that stimuli yielding stronger than anticipated pain would be associated with larger amplitudes of P2 potential.

## Methods

### Subjects and Procedure

Twenty-eight healthy subjects took part in the study. One subject showed signs of skin irritation after the first few laser stimuli, and was withdrawn from the experiment. Three subjects rated very low stimulus intensities as painful during the initial configuration of stimulus intensity and consequently, we could not identify any robust LEPs in their recordings. Thus, the final sample comprised 24 subjects (12 females, 12 males) aged 26.2 ± 3.4 (mean ± SD). All subjects gave their written consents prior to the experiment. The study was approved by the University of Liverpool Research Ethics Committee. Participants received £15 to compensate for their time and travel expenses.

The procedures of the experiment were similar to previous LEPs studies (Schulz et al. 2011; Stancak et al. 2015). Participants were told that we were interested in details of their pain experience associated with arrival of a laser stimulus, and how these change when they are involved in a cognitive task. In half of trials, a laser stimulus was administered while subjects viewed the Rubin vase-face illusion (RVI) (Rubin 1915). Activations seen during spontaneous figure-background reversals in RVI usually remain within the primary and higher order visual areas (Andrews et al. 2002; Hasson et al. 2001; Hesselmann et al. 2008; Ishuzu and Zeki 2014; Kleinschmidt et al. 1998), and can therefore be separated from parallel pain-related cortical activations. Subjects were told that the object would have a form of a white vase on black background or a black vase on white background, and that they might perceive spontaneous flipping of their perception from vase to faces or reverse. The subject’s task was to count the number of figure reversals irrespective of their direction (a vase to faces or vice versa).

While a range of different cognitive tasks have been used as distracters in previous studies [reviewed in Legrain et al. (2012)], RVI was specifically selected as a distracter task in the present study because it requires a continuous attentional focus to a static stimulus, and does not require any motor response which is known to reduce LEPs (Nakata et al. 2004). Since the gaze remains focused to the centre of the visual field and the image of the Rubin figure does not change over trials, the task minimises saccadic eye movements which would also interfere with LEPs.

In the other half of trials, subjects focused on their sensations occurring on their stimulated hand. Subjects were told that, regardless of the identical intensity of the laser stimuli, their sensations were likely to vary across 60 trials.

Laser stimuli were applied to the dorsum of the left hand using an Nd–YAP laser stimulator (Stim1324, El.En., Italy). The pulse duration was 4 ms, and the spot size was 5 mm. The intensity of the laser stimulus was adjusted for each subject individually prior to the first block by incrementing the stimulus intensity from 1.25 J in steps of 0.25 J. The intensity producing a moderate pain sensation rated 5 or 6 on a 10-point rating scale on three successive trials was used throughout. A score of 3 corresponded to the pain threshold.

All visual stimuli were presented on a black screen on a 19-inch LCD monitor having a resolution of 1280 × 1024 pixels. Fifteen different variations of RVI were used. Each of the 15 RVIs appeared twice, once as black figure on a white background, and on a different trial as white figure on black background. The order of the 30 RVI images was randomised. Each RVI, sized 200 × 300 pixels, occupied the centre of the screen.

**Fig. 1:**
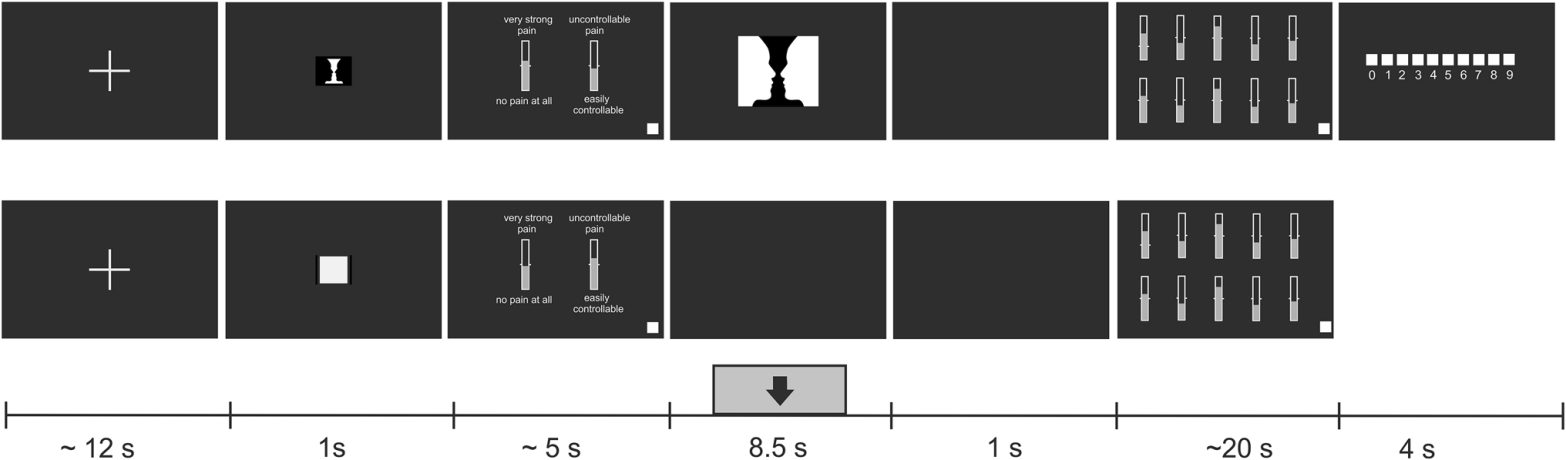
The scheme of trials. Thirty RVI-focus (top row) and 30 hand-focus (bottom row) trials were presented in pseudo-random order. At the beginning of both types of trials, a resting interval with a fixation cross occurred. A miniature RVI figure in RVI-focus trials, or a white square in hand-focus trials were shown as a cue to the type of trial (1 s). Subsequently, subjects indicated their predicted intensity and controllability of expected pain. The two rating scales stayed on the screen until the white rectangle in the lower right corner of the screen was clicked. In RVI-focus trials, a Rubin figure was shown for 8.5 s. In hand-focus trials, subjects viewed a blank screen. A laser stimulus was applied at a random instant within a 4.5 s interval (indicated by the grey rectangle with black arrow) starting 2 s after onset of Rubin figure or a blank screen. This 8.5-s stimulation period was followed by a blank screen for 1 s to allow subjects to experience the full range of sensations triggered by laser stimulus. A set of 10 rating scales was then shown, please see Methods for description of anchors. After evaluating the pain experience using every scale, subjects pressed a white rectangle in the lower right corner to proceed. In RVI trials, subjects indicated the number of figure reversals by clicking on one of the 10 white rectangles representing the range of 0–9 reversals. This screen was shown for a fixed period of 4 s. The total duration of a trial was about 50 s

The structure of RVI- and hand-focus trials is shown in Fig. 1. After displaying a fixation cross, a trial began with a cue of 1 s duration informing the subject about the focus of attention. A small-sized Rubin vase or a white square cued each of the two attentional conditions. In the next phase, two pre-stimulus rating scales were shown. After completing the two pre-stimulus ratings, subjects allocated their full attention to their left hand while viewing a blank screen in half of the trials, or counted the number of figure reversals in RVI condition while still attending to sensations in the other half of trials. Thus, the attention was split between the RVI and the pain monitoring task during the attentional distraction trials. The laser stimulus occurred at a randomly selected time during a 4.5 s period, starting 2.0 s after the pre-stimulus rating scales disappeared from the screen. Each stimulus was followed by a 1 s rest epoch allowing subjects to experience and evaluate any sensation on their hand or elsewhere in the body. Ten post-stimulus scales were then presented. After completing ratings on all 10 scales, subjects pressed a white square located in the lower right corner, which ended the post-stimulus rating period. In RVI trials, a screen showing 10 horizontally aligned squares, labelled from “0” to “9” was displayed for 4 s. Subjects reported the number of figure reversals by selecting the appropriate square using a computer mouse. Each trial, consisting of the fixation cross period, pre-stimulus rating period, stimulation period, post-stimulus rating period, and figure reversal count reporting in RVI trials lasted about 50 s. This long inter-stimulus interval and the method of pointing the laser beam to a different spot on the hand on each trial were implemented to avoid any systematic build up of skin temperature which may occur if laser stimuli are presented to the same area of a white skin at intervals shorter than 30 s (Leandri et al. 2006). A resting period of about 4–5 min was inserted after the 30th stimulus to allow subjects to refresh. During this break, the stimulated hand area was carefully examined for any signs of skin irritation, and the electrode impedances were checked, and individual electrodes moistened if necessary.

Pain and pain-related sensations were evaluated using a number of visual analogue scales. All scales were vertical columns with a white frame and white fill, sized 30 × 200 pixels. Two pre-stimulus scales were plotted next to each other in the centre of the screen. Ten post-stimulus scales were ordered in two horizontal rows each having 5 scales. Subjects pressed the computer mouse button after dragging the cursor to a particular place on each of the scales which best matched a particular aspect of their sensation. Pressing the button was associated with filling the scale column with grey colour to the height of the cursor. The pain intensity scale had a horizontal white tick at 33% of the height of the scale. This value represented the pain threshold. The rest of the scales had horizontal ticks at 50% of scale height. All scale values, read in units of screen pixels, were transformed to range from 0 to 100 using a linear transform in Matlab v. 8.5 (The Mathworks, Inc., USA).

Description of scale anchors and their selection are explained in our previous study (Stancak et al. 2015), except for the attention focus scale which was designed to evaluate the relative strength of attentional focus towards the hand or towards the computer screen on every trial. The post-stimulus scales were as follows: pain intensity (anchors: “no pain at all”–“very strong pain”), pricking sensation (“no pricking sensation”–“very pricking”), burning sensation (“no burning sensation”–“very burning”), warming sensation (“no warming”–“clear warming”), after-sensations sustained in the stimulated region for seconds after laser stimulation (“no after-sensations”–“strong after-sensations”), body sensations in any region of the body outside the stimulated hand area (“no body sensations”–“a lot of body sensations”), and pain unpleasantness (“neutral”–“very unpleasant”), and arousal (“not arousing at all”–“very arousing”). To evaluate the level and direction of attention allocated to the stimulus and associated sensations on the last trial, the attention focus scale was used (“screen attended”–“hand attended”). Finally, one scale measured subjects’ perception of stimulus onset time over the waiting period (“much sooner than expected”–“much later than expected”). The pre-stimulus rating scales addressed expected pain intensity (“no pain at all”–“very strong pain”) and controllability of pain (“easily controllable pain”–“uncontrollable pain”).

The order of the rating scales varied randomly across trials. Subjects were informed that their first response was best, however, that they could change any scale value as many times as they wished. Also, subjects were told to indicate the absence of a particular sensation on a given trial by skipping the appropriate scale. Subjects were allowed to practice filling the pre- and post-stimulus scales until they felt confident about the procedures and meaning of individual scales. The explanation of instructions and the training period lasted about 25 min.

### Recordings

EEG was recorded continuously using the 129-channel Geodesics EGI System (Electrical Geodesics, Inc., Eugene, Oregon, USA) with the sponge-based Geodesic Sensor Net. The sensor net was aligned with respect to three anatomical landmarks including two pre-auricular points and the nasion. The electrode-to-skin impedances were kept below 50 kΩ and at equal levels in all electrodes. The recording band-pass filter was 0.1–200 Hz, and the sampling rate was 1000 Hz. The electrode Cz was used as the reference electrode.

### Analysis of Scalp LEPs

EEG data were transformed to reference-free data using common average reference method (Lehmann 1987). The common average reference method was used to compute reference-free data as this spatial transform allows for subsequent source dipole modelling of evoked potentials. Eyeblink and ECG artefacts were removed from the data using the principal component analysis method (Berg and Scherg 1994) in BESA 6.0 (Megis GmbH, Germany). Further, movement or electrode artefacts were identified visually and excluded from the analysis. The average number of accepted trials was 25.4 ± 1.7 and 26.9 ± 1.4 (mean ± SEM) trials in hand-focus and RVI-focus condition, respectively. Epochs of interest stretched from − 0.3 to 1.6 s relative to the stimulus onset, using the interval of − 0.3–0.0 s as the baseline. LEPs were band filtered from 0.5 Hz (forward-phase, 6 dB/octave) to 40 Hz (zero-phase, 24 dB/octave).

### Exploratory Factor Analysis of Pain Scales

Subjective reports and the variables describing temporal aspects of laser stimuli can potentially be used as predictors in single-trial LEP analysis. However, aspects of the pain experience such as pain intensity and pain unpleasantness are strongly inter-correlated. To ensure that the predictors in the regression model would be relatively uncorrelated and to reduce the large number of variables to a few independent entities in the subjective pain experience, exploratory factor analysis was employed. In factor analysis, a set of correlated variables describing a material object or a subjective phenomenon are transformed to a relatively small number of unobserved, latent variables or factors. The observed variables are modelled by linear combinations of these factors and residual, unexplained variance.

The pre- and post-stimulus scale values, trial order number, and within-trial laser stimulus onset time acquired in 60 trials and 24 subjects (1440 cases) were used to compute one correlation matrix. The multi-collinearity of the correlation matrix, indicative of functionally linked variables, was evaluated using the Kaiser–Meyer–Olkin method and Bartlett test of sphericity in SPSS v. 21 (IBM Corporation, USA). Ones were inserted into the diagonal of the correlation matrix. Principal component analysis was applied during the initial extraction of components, and the component solution was rotated using normalised Varimax rotation to ensure maximum independence of components. The number of components was evaluated using the eigenvalue one criterion and by inspecting the component scree plot. Factor scores larger than |0.3| were interpreted. Interpretation of factors was based on the most salient loadings in each factor, however, variables with very low loadings were also taken into consideration (Gorusch 2008).

To analyse the correlations between LEPs and factors obtained in factor analysis, factor scores were computed on each trial in every subject using the weighted scaling method (Anderson and Rubin 1956). The weighted scaling method evaluates the factor scores in each trial as a sum of products of factor loadings and observed values in variables contributing to a particular factor. This method of factor scores calculation maintains the full variance in the data, hence allows to evaluate effects of attentional focus on subjective factors, and to use the factor scores as predictors in a multiple regression analysis involving select source dipole waveforms.

### Source Dipole Analysis of LEPs

Improvement of signal-to-noise ratio of single-trial evoked responses before performing statistical analysis is an essential step in single-trial analysis (Spencer 2005).

Methods to improve signal-to-noise ratio of single-trial evoked responses include time–frequency or spatial filters, independent component analysis (Huang et al. 2013; Stancak et al. 2015), principal component analysis, or source dipole modelling. Discrete source dipoles represent the topographic and temporal features of one, or a small number of evoked-potential components and therefore provide natural spatial filters for single-trial analysis. A source dipole modelling approach allowed us to attenuate effects of noise and of those generators which did not change in response to attentional task but which overlapped in space and time with the attention-related LEPs changes.

The grand average LEPs, averaged over all subjects and both attentional conditions, were analysed using source dipole analysis in BESA 6.0 program. Equivalent current dipoles (ECDs) were fitted sequentially in the order of peak latencies of individual LEP components evaluated using global field power waveform, similar to previous studies (Hoechstetter et al. 2001; Stancak et al. 2002, 2013). Classical low resolution electromagnetic analysis recursively applied (CLARA) method was used as an independent source localisation method to verify the presence of each ECD. In CLARA, the singular decomposition value cut-off was 0.01%, and the cross-validation error was 1.0. If a small difference (in the range of 10 mm) in the location of an ECD and a corresponding CLARA cluster was encountered, the fitted ECD maximum was preferred in order to maintain the integrity of the source dipole model over the entire LEP epoch. Source dipole modelling assumed a 4-shell ellipsoid head volume conductor model using the following conductivities: brain = 0.33 S/m; scalp = 0.33 S/m, brain = 0.0042 S/m, and cerebrospinal fluid = 1 S/m.

Source dipole waveforms in the hand-focus and RVI-focus conditions were compared statistically using series of Student’s *t* tests which were repeated for each time sample ranging from 0 to 1300 ms. To avoid Type I error due to the large number of tests, P values were computed using a permutation method involving 5000 permutations (Maris and Oostenveld 2007).

The source dipole model was back-projected to original artefact-cleaned continuous EEG data of every subject. This step yielded a relatively small number of source waveforms each representing a continuous signal generated in a particular cortical region. The continuous source waveform data were epoched in the interval ranging from − 300 to 1600 ms relative to the onset of the laser stimulus.

Brain responses to external stimuli vary over time due to both the stimulus-driven, bottom-up processes and top-down modulations, and also due to noise related to e.g., spontaneous endogenous rhythms in physiological systems. Subjective or cognitive performance measures have been employed in the analysis of single-trial evoked potential data to separate noise variance from the meaningful information about the perceptual decisions occurring with each stimulus. Mapping behavioural data onto brain electrical activity requires a statistical model, such as a multiple linear regression model (Ratcliff et al. 2009; Rousselet et al. 2011), support vector machines (Schulz et al. 2012), multivariate decoding based on accumulation of topographic activity (Tzovara et al. 2015), or a linear fixed effects model (Michail et al. 2016). While single-trial analysis of evoked potentials was previously performed using a variety of methods, the multiple regression analysis utilised here fitted the objective to associate multiple aspects of subjective pain experience with the P2 component whilst accounting for possible inter-correlations between predictors.

The single-trial source dipole waveforms, representing a cleaned and focal signal generated in a given cortical region, were used as dependent measures in a multiple linear regression analysis in Matlab v. 8.5 (The Mathworks, Inc., USA) in which the single-trial scores of the factors showing a statistically significant effect of attentional manipulation were used as predictors. The multiple regression analysis was computed using data from clean trials whereby the order numbers of retained trials were used to extract the factor scores and other variables from a complete set of 60 trials available in each subject. The slopes of regression for each predictor variable obtained in every subject were analysed using univariate T-tests. A 95% confidence level was employed.

## Results

### Behavioural Data

The mean intensity of laser stimuli was 2.10 ± 0.43 J (mean ± SD) which corresponded to the mean fluency of 10.5 ± 2.2 J/cm^2^. The mean pain intensity level during the experiment was 43.2 ± 11.5 points, and it varied by an average of 12.9 ± 4.8 points over the course of 60 trials. All subjects showed spontaneous reversals of RVI with an average number of reversals of 3.8 ± 0.3 (mean ± SEM) over the period of 10 s. Individual mean numbers of reversals ranged from 1.6 to 7.5. This data suggests that all subjects experienced RVI, albeit with an individually varying strength of illusion effect.

Table 1 shows the mean values (± SEM) of 10 post-stimulus and 2 pre-stimulus variables, and *t* and the bootstrap-corrected P values obtained from paired Student’s *t* test. The SPSS bootstrapping method involving 2000 permutations was used to correct the P values in order to mitigate the risk of false positive results due to the large number of t-tests. Focusing on RVI compared to focusing on the left hand was associated with decreased ratings in all scales except the perceived stimulus onset time. The statistical significance at a corrected P < 0.05 was reached in pricking sensation, attentional focus, and anticipated pain. Notably, pricking pain and anticipated pain intensity were smaller in the RVI than the hand-focus condition.

**Table 1:** Mean values ± SEMs of 12 visual analogue rating scales, *t* values, and bootstrap corrected P values

	Hand focus	RVI focus	t_(23)_	P
Pain intensity	44.0 ± 2.50	42.4 ± 2.26	1.96	0.084
Pricking sensation	56.8 ± 3.59	54.1 ± 3.65	2.26	0.039*
Unpleasantness	44.2 ± 2.98	42.2 ± 2.78	1.92	0.078
Burning sensations	38.1 ± 3.95	36.6 ± 3.59	1.14	0.290
After-sensations	35.4 ± 4.02	31.8 ± 3.68	2.23	0.091
Perceived stimulus onset	− 2.2 ± 1.89	2.1 ± 1.22	− 0.41	0.972
Arousal	38.8 ± 4.32	36.7 ± 4.09	1.88	0.089
Attentional focus	27.5 ± 3.84	− 28.6 ± 2.62	9.38	0.001*
Body sensations	17.8 ± 3.51	17.2 ± 3.51	2.00	0.105
Warming	38.2 ± 3.12	35.6 ± 2.81	2.00	0.067
Anticipated pain	48.1 ± 2.13	44.4 ± 1.93	3.05	0.008*
Controllability	39.3 ± 3.55	37.9 ± 3.52	0.64	0.57

*RVI* Rubin vase-face illusion

*Statistically significant effect (corrected P < 0.05)

### Factor Analysis of Subjective Responses

To reduce the number of variables to relatively few underlying components, factor analysis was employed. Every variable counted 1440 values (24 subjects × 60 trials). Values of the body sensations scale were not normally distributed due to a large of number zero scale values associated with the lack of any body sensations in 4 subjects. Further, the attentional focus scale showed a bimodal distribution due to an obvious difference between the two attentional conditions. Therefore, these two variables were not included into factor analysis. Thus, factor analysis was computed using 8 post-stimulus rating scales, 2 pre-stimulus scales, physical stimulus onset time, and the trial order number. Inclusion of trial order into factor analysis allowed us to identify and quantify slow changes in pain perception over the course of the experiment, such as habituation or sensitisation. The Kaiser–Meyer–Olkin measure of sampling accuracy of 0.82, and Bartlett’s test of sphericity [χ^2^_(91)_ = 5520.2, P < 0.00001] both indicated the absence of multi-collinearity in the input correlation matrix. Four components explaining 63% of the total variance were extracted. Table 2 shows the component loadings, eigenvalues, and the relative explained variance of the five components. Factor 1 was loaded positively by pain intensity, pricking sensations, arousal, unpleasantness, and moderately by burning sensations and after-sensations. All subjects described the sharp pricking and burning sensation as the hurting component of their pain experience, and reported a burning heat sensation occurring later than the pricking sensation. This factor represents essential aspects of pain.

**Table 2:** Rotated factor matrix, eigenvalues, and explained variance

Factor	Factor 1	Factor 2	Factor 3	Factor 4
Pain intensity	**0.826**	0.246	0.183	0.041
Pricking sensation	**0.809**	− 0.119	− 0.073	0.116
Unpleasantness	**0.810**	0.187	0.030	0.011
Burning sensation	**0.520**	0.128	**0.578**	− 0.055
After-sensations	**0.536**	0.189	**0.568**	− 0.061
Perceived stimulus onset	0.059	0.075	0.072	**0.795**
Arousal	**0.746**	0.242	0.164	− 0.020
Warming	0.078	0.172	**0.742**	0.008
Anticipated pain	0.107	**0.815**	0.102	0.032
Controllability	0.194	**0.805**	0.082	0.018
Stimulus onset time	0.017	− 0.048	− 0.029	**0.778**
Trial order number	− 0.155	− **0.326**	**0.456**	0.155
Eigenvalue	3.995	1.321	1.206	1.135
Variance explained (%)	33.3	11.0	10.05	9.46

Factor loadings larger than |0.30| are highlighted

Factor 2 had positive loadings of anticipated pain intensity and lack of control over upcoming pain, and a negative loading of trial order number. The negative loading of trial order number suggests that anticipated pain linearly decreased over the course of the experiment, possibly as a part of a habituation process or learning.

Factor 3 was loaded positively by warming sensations, after-sensations, and burning sensations. All subjects reported continuing warming or burning sensations over the stimulated hand area, which evolved from the previous burning sensation and lasted for seconds. Nine subjects also reported prickling or tingling sensations, especially if the initial sensation was a sharp pricking pain. These sensations were all represented by a single after-sensation scale. None of the subjects labelled after-sensations as painful. Thus, Factor 3 refers to the warming component of sensory experience, featuring primarily continuing, non-painful warming evolving from previous burning sensation, and possibly other less consistent after-sensations.

Factor 4 was loaded positively by stimulus onset time and expected onset time, and it is denoted further as the stimulus onset time component. The component loadings, and a statistically significant pair-wise correlation coefficient between perceived and physical onset times [r(1338) = 0.27, P < 0.001] suggested that subjects were able to accurately capture the onset time of laser stimuli even in the presence of attentional distraction.

Table 3 gives mean values ± SEM in the hand-focus and RVI focus condition, and *t* and corrected P values obtained from paired t-tests. Factor 1 representing essential aspects of pain, and Factor 2 accounting for anticipated pain and lack of control over the pain showed smaller mean values in RVI than the hand-focus condition (corrected P < 0.05).

**Table 3:** Mean values ± SEMs of factor scores in the hand-focus and RVI-focus condition, T values, and bootstrap corrected P values

	Hand focus	RVI focus	t_(23)_	P
Factor 1	196.0 ± 12.8	185.3 ± 11.9	2.59	0.017*
Factor 2	99.8 ± 6.8	93.3 ± 6.3	2.32	0.035*
Factor 3	104.2 ± 7.5	97.9 ± 6.8	2.50	0.050
Factor 4	13.1 ± 1.88	12.3 ± 6.1	0.486	0.621

*RVI* Rubin vase-face illusion

*Statistically significant effect (corrected P < 0.05)

Results suggest that attentional distraction attenuated all essential aspects of pain, in particular the pricking sensation. Further, subjects anticipated less pain in RVI than hand-focus trials. Anticipated pain intensity and lack of control over the pain were associated with the trial order number in Factor 2, suggesting that subjects might have learned the association between RVI and a decreased level of pain and showed a progressive decrease of anticipated pain over the course of experiment.

### Attention Effects on Averaged LEPs

**Fig. 2:**
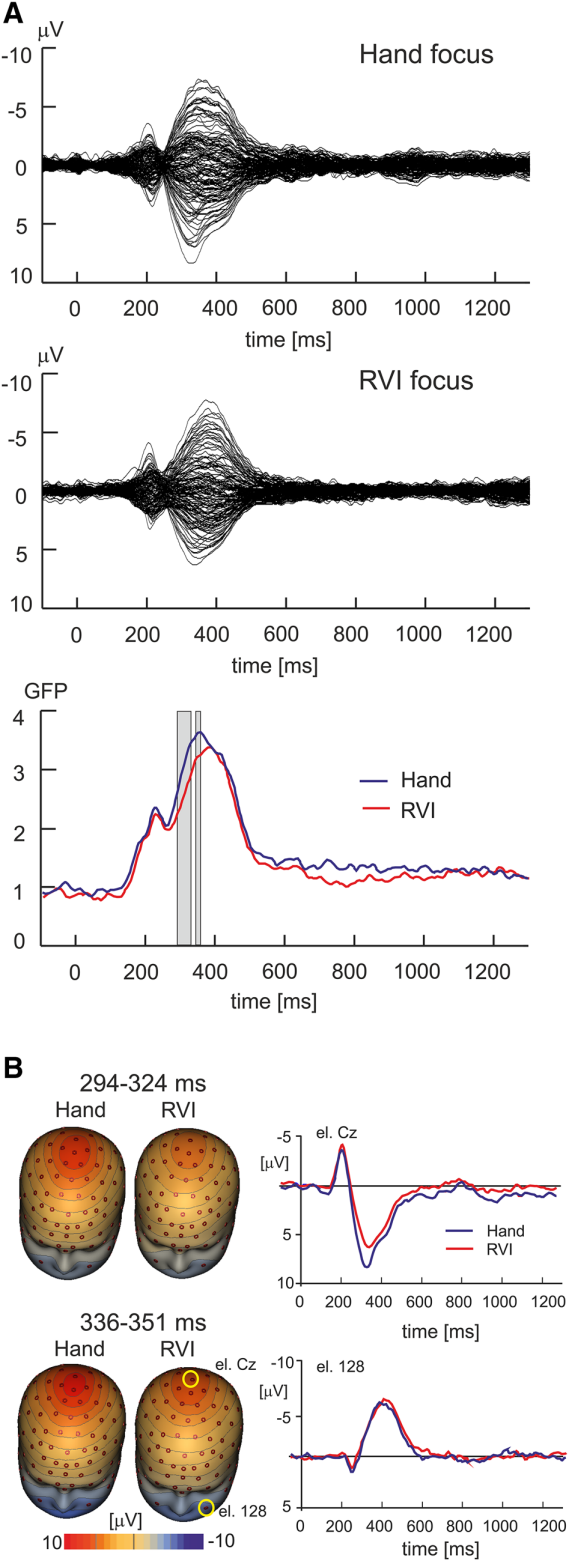
Effects of attentional focus on LEPs. **a** The top two panels show the butterfly plots of grand average LEPs in the hand-focus and RVI-focus condition. The bottom panel illustrates the global field power in both conditions. The two vertical strips correspond to two latency epochs (294–324 and 336–351 ms) manifesting a statistically significant effect of attention focus (corrected P < 0.05). **b** Topographic maps of LEPs in RVI and focused attention in intervals 294–324 and 336–351 ms (left panel), and LEP potentials at the vertex electrode Cz and at a lower face electrode 128 (right panel). Two yellow circles in the lower right map highlight electrodes Cz and 128

Figure 2a shows the butterfly plots of grand average LEPs and global field power in the RVI- and hand-focus condition. The butterfly plots illustrate the distinct LEP components N1, N2, P2, and N3/P2. The N3/P2 corresponds to the late part of the P2 component in the latency range > 350 ms in previous studies (Legrain et al. 2003, 2009a). The N3 label highlights a different topographic pattern over the central region of the scalp (Hu et al. 2014; Stancak and Fallon 2013) and in the forehead and facial regions (Stancak and Fallon 2013; Stancak et al. 2013) compared to the early P2 component.

The global field power in the interval from − 100 to 1300 ms in both attention conditions were compared using paired t-test involving a permutation method with 5000 permutations to control for multiple tests. Two intervals showed a statistically significant difference between the two attention conditions: 294–324 and 336–351 ms. Both intervals fell into the latency period of P2 and N3/P2 components which are featured by a distinct positive potential at the vertex and a negative potential over face electrodes. Figure 2b shows the grand average topographic maps of LEPs in both intervals and LEPs in two electrodes, one located at the vertex (Cz) and another in the left lower face (electrode 128). The positive vertex potential was larger in the hand-focus than RVI-focus condition both in the 294–324 ms [t(23) = 3.61, P = 0.002] and 336–351 ms [t(23) = 4.56, P < 0.001] latency interval. In contrast, the negative potential in the lower face, illustrated at electrode 128 in Fig. 2b, did not show a statistically significant difference between the two attention conditions either in the 294–324 ms [t(23) = − 0.63, P = 0.53] or 336–351 ms [t(23) = − 0.53, P = 0.60] interval.

### Attention Effects on Cortical Sources of LEPs

**Fig. 3:**
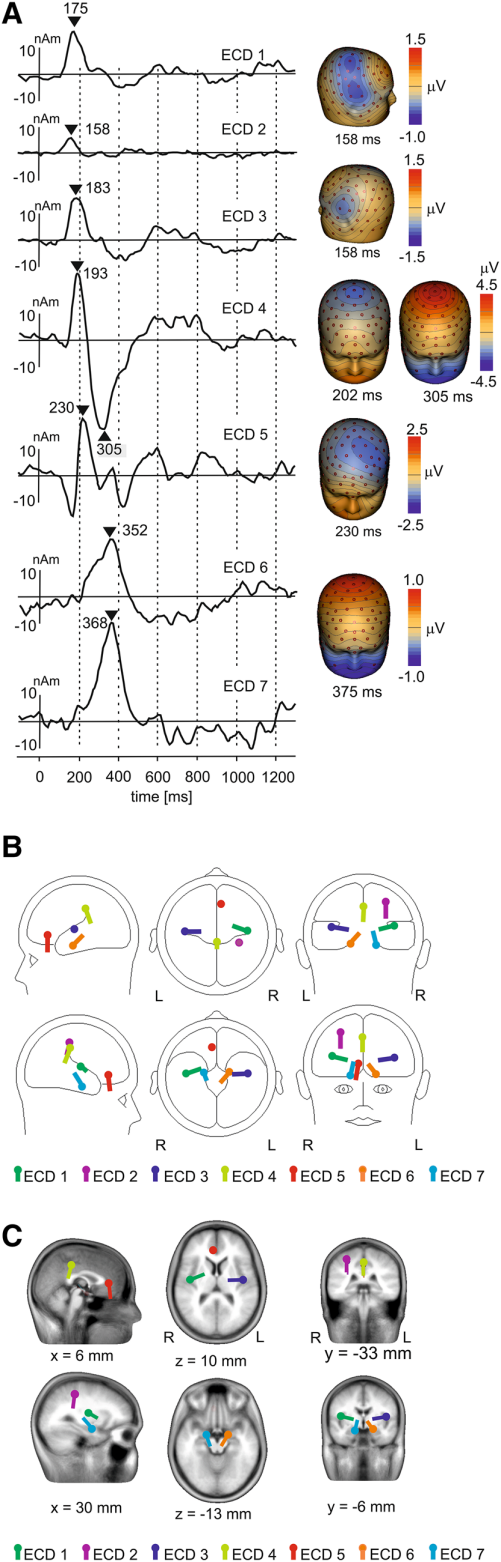
Source dipole model of LEPs. **a** Source dipole waveforms in seven ECDs fitted to grand average LEPs (left panel). In each of seven ECDs, the peak latencies and the topographic maps of LEPs (right panel) are shown. **b** Locations and orientations of seven ECDs in the schematic glass brain. **c** Locations and orientations of ECDs in the standard anatomical MR image of the brain

To improve the signal-to-noise ratio of single trial LEPs, LEPs were modelled by a set of equivalent current dipoles (ECDs) in BESA 6.0. This step allowed us to quantify the amplitude variations of the attention-sensitive P2 component whilst eliminating impacts of the cortical sources which contributed to the overall amplitude of P2 but did not encode effects of the attentional task. The spatial LEP patterns used to construct the source dipole model, and the source dipole waveforms are shown in Fig. 3a. Locations of ECDs are shown in a glass brain and in the standard brain subject in Fig. 3b, c, respectively.

The first ECD was fitted based on the negative potential maximum at the right temporal region during the initial part of the N1 component at 158 ms. Although the N1 potential over temporal electrodes continued to peak later at 175 ms, the N1 component was dominated by the strong N2 potential which masked the weaker N1 potential. The first ECD was a radial dipole located in the right operculo-insular cortex (approximate Talairach coordinates: x = 42 mm, y = − 11 mm, z = 12 mm). The negative maximum over the right temporal scalp region was associated with another negative maximum over the right central electrodes during the early part of the N1 component. This potential component was modelled with ECD2 located in the right primary somatosensory cortex (Brodmann area 2, approximate Talairach coordinates: x = 30 mm, y = − 34 ms, z = 45 mm). ECD2 peaked at 158 ms. During the initial part of the N1 component, another negative potential maximum occurred over the left temporal scalp region. This ipsilateral N1 potential was modelled by ECD3 which fitted into the left operculo-insular cortex (approximate Talairach coordinates: x = − 42 mm, y = − 11 mm, z = 11 mm). ECD3 peaked at 183 ms. The N2 potential, peaking at 198 ms and showing a distinct negative maximum over the vertex, was fitted by ECD4 located in the posterior cingulate cortex (PCC) (Brodmann area 23/31, approximate Talairach coordinates: x = 1 mm, y = − 33 mm, z = 39 mm). ECD4 had a predominant radial orientation and pointed slightly anterior towards the Cz electrode. Therefore, it is likely that ECD4 also picked activation from the regions lying along the course of the dipole, i.e. the adjacent dorsal mid-cingulate cortex and supplementary motor area. ECD4 showed later a strong positive maximum at 320 ms which contributed to the positive P2 component.

The N2 and P2 potential components were separated in the overall strength of the potential field evidenced by a dip in global field power as shown in Fig. 2b. This period of a comparatively weak electrical activity showed a negative potential maximum over the left and midline frontal electrodes at 220 ms. It was modelled by a predominantly radial ECD5 with an origin in the rostral anterior cingulate cortex (Brodmann area 24, approximate Talairach coordinates: x = 6 mm, y = 31 mm, z = 6 mm).

Finally, the N3/P2 potential complex was featured by the negative potential field over the left and right lower face, and a positive potential in the midline parietal electrodes. This potential configuration suggests one or two symmetrically located dipoles in the depth of the brain. Both free fitting at the latency points 350–360 ms and CLARA pointed to the presence of two source dipoles labelled ECD6 and ECD7. ECD6 was located in the left medial temporal cortex (Brodmann area 34, approximate Talairach coordinates: x = − 17 mm, y = − 5 mm, z = − 14 mm). ECD7 was located in the right medial temporal cortex (Brodmann area 34, approximate Talairach coordinates: x = 17 ms, y = − 5 mm, z = − 14 mm).

Subsequent latency components (> 500 ms) were contributed by the slow oscillatory-like waves seen in ECD1, ECD3, ECD5 and ECD6. Attempts to fit a dipole in this long latency range did not reduce the residual variance, and yielded an ECD outside the boundaries of the head. The 7-dipole model explained 92% of variance in the interval ranging from 0 to 1300 ms.

**Fig. 4:**
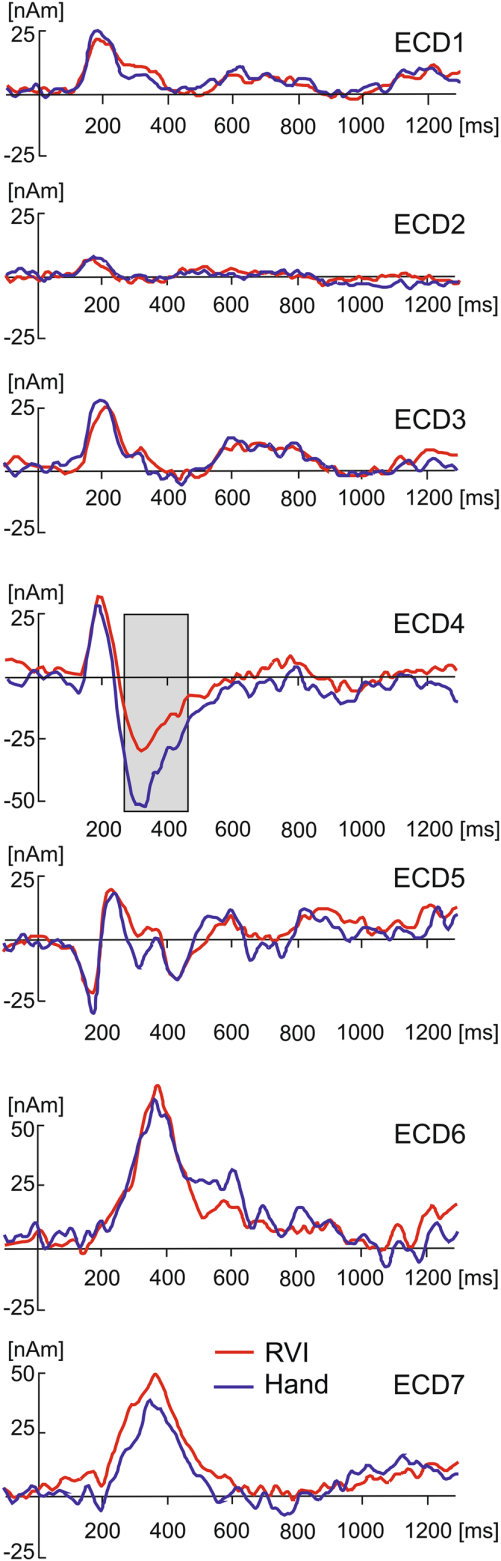
Grand average source dipole waveforms in source dipoles labelled ECD1-ECD7 in two attentional conditions. The grey strip in ECD4 indicates the interval 244–432 ms during which the source activity was stronger in hand-focus than RVI-focus condition at corrected P < 0.05

The 7-dipole source model was used to quantify the source waveforms in every subject in both attentional conditions. The amplitude differences in source waveforms between the RVI-focus and hand-focus condition occurring over the time interval from 0 to 1300 ms were evaluated on each time sample using a paired t-test. Due to the large number of tests, the P values were corrected using the permutation analysis involving 5000 permutations (Maris and Oostenveld 2007). Figure 4 shows the grand average waveforms in the hand-focus and RVI-focus condition. The only source dipole manifesting effect of attention was ECD4. The source activity of ECD4 in the interval 244–432 ms, covering the latency period of P2 potential, was stronger in hand-focus than RVI-focus condition at a corrected significance level of P < 0.05. Since the positive pole of ECD4 pointed to the vertex, the difference between both attention conditions was consistent with a stronger positive potential over the vertex (P2) in hand-focus than RVI-focus condition (Fig. 2b).

### Multiple Regression Analysis

To analyse whether attention-related amplitude changes in amplitudes of the P2 component, represented by ECD4, would be associated with variations in subjective pain experience, the level of visual task engagement, and allocation of attention to the hand or to the task, we conducted a multiple linear regression analysis. The multiple linear regression analysis involved single-trial ECD4 amplitudes as a dependent measure, and single-trial Factor 1 (essential aspects of pain) and Factor 2 (anticipated pain intensity and controllability of pain) scores, and the number of reported figure reversals as predictors. Finally, the attentional scale values, measuring allocation of attention towards the hand or towards the visual stimulus, were also used as a predictor.

**Fig. 5:**
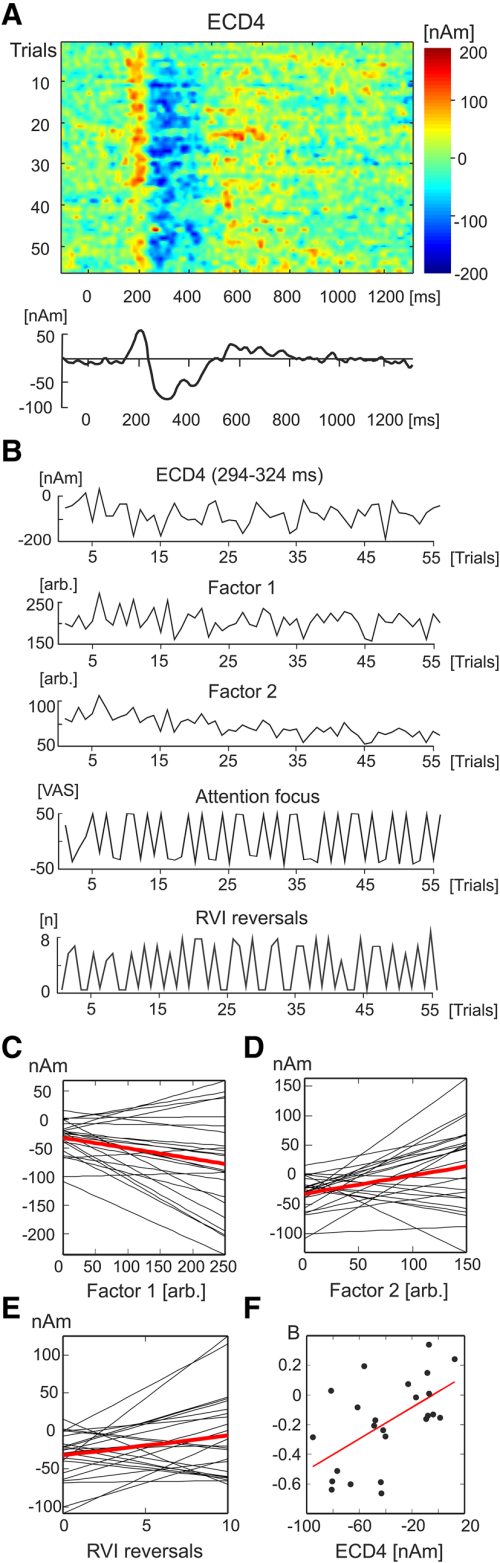
Multiple linear regression analysis of ECD4. **a** Colour-coded single-trial ECD4 waveforms and the average ECD4 waveform in subject S01. **b** Single trial amplitudes of ECD4 in the latency interval 294–324 ms, Factor 1, Factor 2, the strength of attentional focusing towards the hand or RVI, and number of RVI reversals in subject S01. **c** Individual (black lines) and grand average (red line) linear regression lines representing associations between single-trial amplitude variations of ECD4 and Factor 1. **d** Individual and grand average regression lines representing associations between amplitudes of ECD4 and Factor 2. **e** Individual and grand average regression lines for the number of RVI reversals. **f** The scatter plot and the regression line showing the correlation between the individual average strength of ECD4 and the regression slope coefficient computed between amplitudes of ECD4 and Factor 1

Figure 5a illustrates the single-trial variations of ECD4 waveforms in one subject. The mean source dipole moments in the interval 294–324 ms of this subject are plotted in Fig. 5b along with single-trial values in Factor 1 and 2, and the attention focus scale values, and number of RVI reversals. The attention focus values often swung between scores of 50 and − 50 corresponding to a complete hand and RVI focus, respectively.

The multiple regression analysis was carried out separately in every subject using as the dependent measure the average source activity in ECD4 in the latency interval from 294 to 324 ms in which the P2 component showed both the largest amplitude and the largest difference between the two attentional conditions. The univariate t-tests of individual regression coefficients showed that Factor 1 was negatively correlated with ECD4 [t(23) = − 3.08, P = 0.005], and positively with Factor 2 [t(23) = 2.86, P = 0.009] and the number of RVI reversals [t(23) = 2.20, P = 0.039]. The individual and the grand average linear regression lines for each of the three predictors showing statistically significant correlations with single-trial changes in ECD4 are shown in Fig. 5c–e.

The variance explained by a particular regression model was evaluated using R^2^ method in every subject. The regression model involving four predictors explained 5–32% of variance in individual subjects suggesting that trial-by-trial amplitude variations in ECD4, representing the strength of the P2 component of LEPs, were strongly affected by other factors not directly related to the attentional task. Six subjects out of 24 showed a weak positive slope of regression between the ECD4 amplitude and Factor 1. These six subjects appeared to have smaller levels of ECD4 amplitude (− 18.6 ± 13.4 nAm, mean ± SD) than the subjects showing a negative slope of regression (− 45.8 ± 6.8 nAm) which difference reached borderline statistical significance [t(22) = 1.94, P = 0.069]. However, the strength of the negative slope of the Factor 1-ECD4 regression correlated with the average individual amplitude levels of ECD4 [r(23) = 0.57, P = 0.004] (Fig. 5f) suggesting that the differences in individual slopes of regression lines were related to the average strength of source activity during the P2 latency interval.

To explore further the association between Factor 2, involving anticipated pain intensity, and the amplitude of the P2 component, we tested the possibility that the relationship involved a comparatively smaller pain intensity prediction error in trials in which subjects anticipated a high level of pain. Therefore, the intensity prediction error was calculated in every trial as the difference between anticipated and experienced pain intensity. The pain intensity prediction error was not different in the RVI- and hand-focus condition [t(23) = 1.21, P = 0.24]. However, the prediction error correlated with single-trial ECD4 amplitudes representing the strength of the P2 component [t(23) = 2.56, P = 0.018] in a multiple linear regression analysis in which it was used as a predictor together with the attentional focus scale values and the number of RVI reversals. The positive sign of the regression slope was related to the comparatively small values of the intensity prediction error (perceived pain stronger than anticipated pain) in trials with large and negative source dipole moments values.

We also evaluated the strength of correlation in the long latency interval ranging from 244 to 432 ms which showed a statistically significant difference between the hand-focus and RVI-focus conditions in ECD4, which modelled the P2 component of LEPs. The regression effects were similar to those seen in the narrow latency interval of 294–324 ms for Factor 1 [t(23) = − 2.34, P = 0.029] and for the number of RVI reversals [t(23) = 2.63, P = 0.015]. However, the regression coefficients in Factor 2, accounting for anticipated pain intensity and pain controllability, were not statistically different from zero [t(23) = 1.87, P = 0.075] suggesting that effects of Factor 2 were limited to the latency period manifesting the strongest amplitude of P2 component.

Notably, the negative associations between Factor 1, representing essential aspects of pain, and the source strength in ECD4 were consistent with a positive association between the strength of experienced pain and the scalp P2 potential because the negative signs in the source waveform signals point towards the positive part of scalp potential field. Conversely, the positive associations seen to occur between Factor 2 or the number of RVI reversal and ECD4 indicate that large values in these two variables were paralleled with small amplitudes of P2 potential.

## Discussion

Results show, in accord with previous studies, that the positive LEP component at vertex electrodes in the latency window around 300 ms, known as the P2 potential, differentiated attentional distraction from focused attention. Although multiple cortical regions contributed to LEPs during this latency interval, only one source dipole located in the posterior region of the cingulate cortex encoded effects of attentional distraction. Single-trial variations in subjective pain experience encompassing primarily the pain intensity and pricking sensation correlated with the instantaneous amplitudes of the P2 component during manipulation of attentional focus. Further, Factor 2, accounting for anticipated pain intensity and pain controllability, and the number of RVI reversals correlated with the single-trial amplitude variations of the P2 component, suggesting that reorienting attention following a noxious stimulus was modulated by both the anticipated pain and the degree of engagement in the concurrent cognitive-perceptual task.

### Attentional Distraction and LEPs

The P2 component of LEPs in the latency interval from 294 to 350 ms was smaller during the RVI-focus than hand-focus conditions. The amplitude decrease of the P2 component during attentional distraction accords with a number of previous studies (Beydoun et al. 1993; Boyle et al. 2008; Franz et al. 2015; García-Larrea et al. 1997; Kanda et al. 1996; Ohara et al. 2004; Schlereth et al. 2003; Siedenberg and Treede 1996). The present study has not shown any statistically significant effects of attentional distraction in N1 or N2 components which have been reported earlier during attentional distraction (Franz et al. 2015; Friederich et al. 2001) or attentional oddball (Legrain et al. 2002) studies. Absence of N1 and N2 component changes may be related to a comparatively weak oddball component in our task. The stimuli occurred predictably within a relatively short time interval and stimuli were of identical physical qualities.

### Reorienting Attention Towards Noxious Laser Stimulus

The multiple regression analysis of single-trial P2 responses using subjective factors and the number of RVI as predictors sheds new light on the functioning of reorienting attention towards the pain purportedly involving dorsal PCC and posterior part of mid-cingulate cortex. While prevailing LEP studies localised the source of N2 and P2 components in anterior or mid-cingulate cortex (Garcia-Larrea et al. 2003), several studies reported a source of these potential components in the posterior cingulate cortex (Bentley et al. 2003, 2001; Boyle et al. 2008; Bromm 2004; Stancak and Fallon 2013). Notably, a recent intra-cerebral study also showed sources of LEPs in the PCC (350 ms), although these sources occurred less frequently than those in the mid-cingulate cortex (242 ms) (Bastuji et al. 2016). Sources of LEPs peaking at different latency points have been demonstrated in the peri-genual cingulate (220 ms), mid-cingulate (275 ms) and posterior cingulate (290 ms) cortex (Stancak and Fallon 2013). It is likely that the source in the PCC became prominent due to the presence of an attentional task.

The attentional switching appears to involve three distinct associations. In the first association, the strength of the P2 component is associated with variations in perceived pain intensity and other aspects of subjective pain experience such as pricking sensation or unpleasantness. An association between the amplitude of P2 and pain intensity has been established in previous studies involving a non-pain task such as attentional distraction or motor readiness (Boyle et al. 2008; García-Larrea et al. 1997; Stancak et al. 2012). As data suggest that PCC has contributed to attention-related changes of the P2 component, it is noteworthy that PCC has one of the largest representations of GABA-A receptors in the cortex (Palomero-Gallagher and Zilles 2009), and shows patches of opioid receptors (Vogt and Vogt 1999). Thus, it is possible that PCC via connections with anterior mid-cingulate and the supra-genual anterior cingulate cortex (Vogt et al. 2006) may modulate the central nociceptive processing during attentional distraction in either a facilitatory or inhibitory manner.

The second association is featured by the negative correlation between the number of RVI reversals and the amplitude of the P2 component in such a way that the amplitude of the P2 component was smaller in trials with a large number of RVI reversals. Although a few early studies failed to find an association between the difficulty of a distraction task and pain reduction [reviewed in Eccleston and Crombez (1999)], our finding is consistent with a previous LEP study demonstrating a decreased amplitude of P2 to rare stimuli in an oddball paradigm. When subjects were distracted with a visual task requiring a reaction time response, the amplitude of the P2 response to rare stimuli was decreased if the visual-motor task was difficult (Legrain et al. 2005). The analgesic effects of an immersive virtual reality task have been shown to be enhanced by increasing the level of immersion into virtual reality environment using more elaborate helmets (Hoffman et al. 2006). Although the spatial resolution of source dipole analysis does not exclude the role of dorsal mid-cingulate cortex in single-trial changes of the P2 component in the present study, the role of PCC in attentional modulation of pain seems probable considering the importance of this cortical region in default mode network. The default mode network, in which PCC is one of the strongest components, was activated more if subjects let their mind wander away from pain (Kucyi et al. 2013), and in those participants who are likely to engage in a cognitive task rather than focus on their pain (Erpelding and Davis 2013). Single neurons in PCC in macaques have been shown to encode the level of engagement for an attentional task and their activities inversely correlated with performance in an attentional task (Hayden et al. 2009). It appears that a strong engagement into a perceptual task favours a strong resting activation of PCC which may be difficult to sway towards a noxious stimulus in a phasic manner. This phenomenon may explain the negative association between the number of RVI reversals and single-trial amplitudes of P2 in the present study.

The third association of variables in the switching of attention involves effects of anticipation which manifested in the negative correlation between the amplitude of ECD4, representing the P2 component of LEPs, and Factor 2 (anticipated pain intensity and controllability). Notably, the amplitude of ECD4 responses was comparatively small in trials in which subjects anticipated strong pain and perceived a reduced capacity to control the pain. Anticipated pain intensity and uncontrollability of pain were negatively associated with the trial order number suggesting the presence of perceptual learning prompting subjects to anticipate less pain as the experiment progressed. Results suggest that attentional switching is modulated by top-down processes such as anticipation. This finding accords previous behavioural data showing that manipulation of the threat value of a distracter affected the interruptive effect of pain on cognitive processing (Crombez et al. 1998).

Notably, the reward prediction error in a decision task, manifesting in feedback-related negativity, operates during the latency period from 250 to 350 ms (Walsh and Anderson 2012) which overlaps with the latency period of the P2 component. Feedback-related negativity shows a spatial maximum at centro-parietal midline electrodes (Gehring and Willoughby 2002), and some source localisation studies pointed to a source of feedback-related negativity being in PCC (Doñamayor et al. 2011; Müller et al. 2005; Nieuwenhuis et al. 2005). More specifically, the intensity prediction error in the present study, although not different in the distraction and focused attention conditions, bore a positive association with the amplitude of the P2 component. Firing of posterior cingulate neurons has been shown to encode deviation of a chosen option from a standard option in a variety of decision tasks irrespective of values of the chosen option (Heilbronner et al. 2011). Therefore, we speculate that the association between Factor 2 (anticipated pain intensity and pain controllability) and the strength of the P2 component during attentional distraction entails calculation and implementation of intensity prediction error. This hypothesis accords with the finding of a feedback negativity potential during an unexpected omission of pain stimulus which was similar in latency and topographic map to the negative potential associated with a monetary loss (Talmi et al. 2013).

Only 5–32% of variance of single trial ECD4 amplitudes was explained by pain- and task-related variables. Thus, instantaneous P2 responses appear to be modulated by other factors than those accounted for in the present study. Although elucidation of these additional factors contributing to the amplitude variations of the P2 component was beyond the scope of the present study, we speculate that the unexplained variance of P2 amplitude may be related to the role of the posterior cingulate cortex in maintaining the resting state brain activity. For instance, PCC shows the strongest glucose metabolism and oxygen consumption of all cortical areas at rest, and it is one of core regions of the default mode network (Laird et al. 2009; Raichle et al. 2001). PCC activation is not abolished or significantly reduced when the brain engages into a passive perceptual task (Shulman et al. 1997) such as viewing of alternating checkerboard patterns (Greicius et al. 2003). Interestingly, single neuron recordings in macaques showed a suppression of unit activities in PCC during an attentional task, comparable with the deactivation of the default mode network, which was interrupted with bursts of activation during significant parts of the attentional task (Hayden et al. 2009); the phasic increases in firing of single neurons in PCC were not correlated with a tonic suppression of PCC neurons. The strength of basal PCC activation and the spontaneous variations in the default mode network activation may render a comparatively small and variable responsiveness of PCC to external stimuli. Thus, the baseline level and variation in the cortical regions contributing to the P2 component may contribute to a large unexplained variance in the amplitudes of single-trial P2 potentials in the present study. It should be pointed out that uncontrolled spontaneous increases or decreases in skin temperature could also contribute to the unexplained variability in amplitudes of P2 responses. Previous studies have shown that cooling of the skin increased (Green and Akirav 2010) and warming the skin decreased (Churyukanov et al. 2012) the nociceptive thresholds. Future studies should address the role of spontaneous variations in skin temperature on variability of single-trial LEPs.

Present data link the variations in the amplitude of the P2 component during attentional distraction with pain intensity and other aspects of pain experience. This finding adds to the sparse data on correlations of amplitudes of P2 with pain unpleasantness (Boyle et al. 2008) and pain intensity (García-Larrea et al. 1997) during attentional distraction.

The attentional distraction task entailed focusing on a distracter of a different sensory modality to pain, which bore no specific temporal or spatial associations to the noxious stimulus. Recent studies showed that the relative locations of a noxious stimulus and a visual stimulus in peri-personal space affected the temporal order judgment of visual stimuli (De Paepe et al. 2017), and vice versa, the visual cues occurring next to the spot on the hand receiving a noxious stimulus improved the temporal order judgments irrespective of the postures of the hands (De Paepe et al. 2015; Filbrich et al. 2017). This inter-modal, spatial disparity aspect of attentional distraction was beyond the scope of the present research which could represent a limitation.

Our findings provide a neurophysiological underpinning for pain relief observed in procedural pain in acute pain patients, such as patients with burn injury (Hoffman et al. 2011). Results suggest that sharp increases of pain during therapeutic procedures may be better tackled by a distraction task than long-lasting background pain since it was the pricking but not the warming-burning component of pain and after-sensations that were attenuated during attentional distraction. Further, our finding of an association between the amplitude of P2 component and the number of figure reversals in the visual-illusion task accords with the previously reported increased analgesic effect of attentional distraction in a virtual reality task if the feeling of presence in virtual reality was intense (Hoffman et al. 2004b). Finally, results point to the importance of prior information about intensity of impending pain during distraction analgesia, as anticipated pain intensity contributes to the amplitude changes of P2 component, possibly via pain intensity prediction error.

To conclude, our results suggest that reorienting attention towards pain during attention distraction operates in the posterior region of the cingulate cortex during a latency period overlapping with the P2 component of LEPs. We newly show that the attentional switching towards a noxious stimulus in the presence of distraction involves three independent groups of associations: (1) a pain experience modulator which manifests in the linear coupling between the strength of pain experience, in particular the pricking sensation, and the amplitude of P2 component. This association may account for the diminution of the P2 component during attentional distraction. (2) An absorption-engagement association which manifests in the strength of immersion in the cognitive-perceptual task and which is inversely related to the strength of P2 component. (3) An anticipation-related association manifesting in comparatively small amplitude of the P2 source component in trials in which subjects expect a high and uncontrollable pain. These three associations may operate independently or in concert to shape instantaneous pain experience during attentional distraction.
